# The interaction of physical activity and sleep quality with depression and anxiety in older adults

**DOI:** 10.3389/fpubh.2025.1674459

**Published:** 2025-10-23

**Authors:** Yuqing Yuan, Wenying Huang, Chang Hu, Wen Zhang

**Affiliations:** Physical Education College, Jiangxi Normal University, Nanchang, China

**Keywords:** older adults, physical activity, sleep quality, depression, anxiety

## Abstract

**Background:**

With the increase in the global aging population, the mental health of older adults has become increasingly prominent. This study aims to explore the relationship between the interaction of physical activity and sleep quality in older adults and depression and anxiety.

**Methods:**

A multi-stage stratified random sampling method was employed to survey various communities within Nanchang City, collecting data from 2,497 older adults. The assessment tools included the Physical Activity Rating Scale (PARS-3), Pittsburgh Sleep Quality Index (PSQI), the 9-item Patient Health Questionnaire (PHQ-9), and the 7-item Generalized Anxiety Disorder scale (GAD-7).

**Results:**

Among 2,497 participants, 582 (23.30%) had depressive tendencies; 1,122 (44.90%) had anxiety tendencies. The detection rate of depression and anxiety was higher in females than in males. Logistic regression analysis results showed that low physical activity was positively correlated with depression and anxiety (OR = 9.46; 3.66), while poor sleep quality was positively associated with depression and anxiety (OR = 2.40; 9.96) (*P* < 0.05). There is an interactive effect between physical activity and sleep quality in relation to anxiety and depressive symptoms in older adults. Specifically, compared to low physical activity combined with poor sleep quality, the combination of high physical activity and good sleep quality is associated with reduced levels of both anxiety and depression (*P* < 0.05).

**Conclusion:**

This investigation underscores the dynamic interplay among physical activity, sleep quality, depressive symptoms, and anxiety in older adults, forming a critical foundation for designing tailored interventions to enhance mental health outcomes in this population.

## 1 Introduction

The global trend of aging continues to accelerate. Demographic data reveal that 14.6% of Southeast Asia's population comprises adults aged 65 or older. Concurrently, China ranks among nations experiencing the most rapid population aging globally (1). Within this older adult cohort, depression and anxiety represent highly prevalent mental health challenges (2). Empirical evidence indicates that clinically significant depressive manifestations affect approximately one-fifth to one-third (20%-30%) of older adults in China. Concurrently, anxiety-related symptomatology is observed in roughly one-tenth to one-sixth (10%−15%) of older adults (3). Extensive research has established that late-life depression and anxiety frequently co-occur with somatic comorbidities. These conditions notably contribute to autonomic dysregulation, compromise cognitive functions (particularly memory retention and information processing), and significantly elevate the risk of suicidal ideation or behavior among older adults (3–5). Compared to non-depressed peers, older adults with depression exhibit significantly greater mortality risk. Furthermore, depression directly impairs cardiovascular integrity and disrupts metabolic homeostasis (6, 7). Consequently, proactive clinical prioritization of geriatric psychological wellbeing is imperative.

Physical activity (PA) has been established as an effective emotional regulation strategy, modulating affective states through both physiological (8–10) and psychological (11–13) pathways. According to the tenets of cognitive-behavioral theory (14), human cognition, emotion, and behavior are interconnected. Robust evidence confirms that physical activity ameliorates depressive and anxiety symptoms in geriatric populations. The principal neurobiological mechanism entails the regulation of neuroendocrine activity, prompting the endogenous secretion of compounds such as endorphins that elicit euphoric states (15, 16). Concurrently, physical activity attenuates peripheral inflammatory processes. This anti-inflammatory action attenuates disturbances in neurochemical homeostasis, including impaired neurotransmitter signaling, and suppresses excessive activation of the hypothalamic-pituitary-adrenal (HPA) axis, which serves as a central pathway mediating the neuroendocrine stress response (17–19). Moreover, physical activity directly induces neuromuscular relaxation, alleviating somatic tension and affective distress (20–22). Crucially, nocturnal rest quality exerts profound modulatory effects on emotional states (23, 24). Suboptimal sleep constitutes a significant independent predictor for depressive and anxiety disorders, with epidemiological evidence consistently demonstrating substantially elevated depression prevalence among individuals with sleep disturbances compared to age-matched counterparts (25–27). The International Society for Sleep Research (ISRS) emphasizes that nocturnal restoration processes involve active cerebral maintenance and neural homeostasis (28). Consequently, optimal sleep architecture promotes emotional stability and confers prophylactic effects against affective disorders (29, 30).

Prior research has established that physical activity exerts a significant impact on depression and anxiety, while sleep quality is similarly associated with these mental health conditions. Nevertheless, the interplay or synergistic effects of physical activity and sleep quality on depressive and anxiety symptoms remain underexplored, especially among older adults in China. Consequently, this study aims to examine the interactive effects of physical activity and sleep quality on depression and anxiety among Chinese older adults. Rather than evaluating these factors in isolation, the analysis centers on their interaction within China's distinctive sociocultural and epidemiological milieu. This approach yields novel etiological insights that can inform the development of precision, culturally calibrated interventions aimed at reducing affective morbidity in this rapidly aging population.

## 2 Materials and methods

### 2.1 Participants

**Figure 1:**
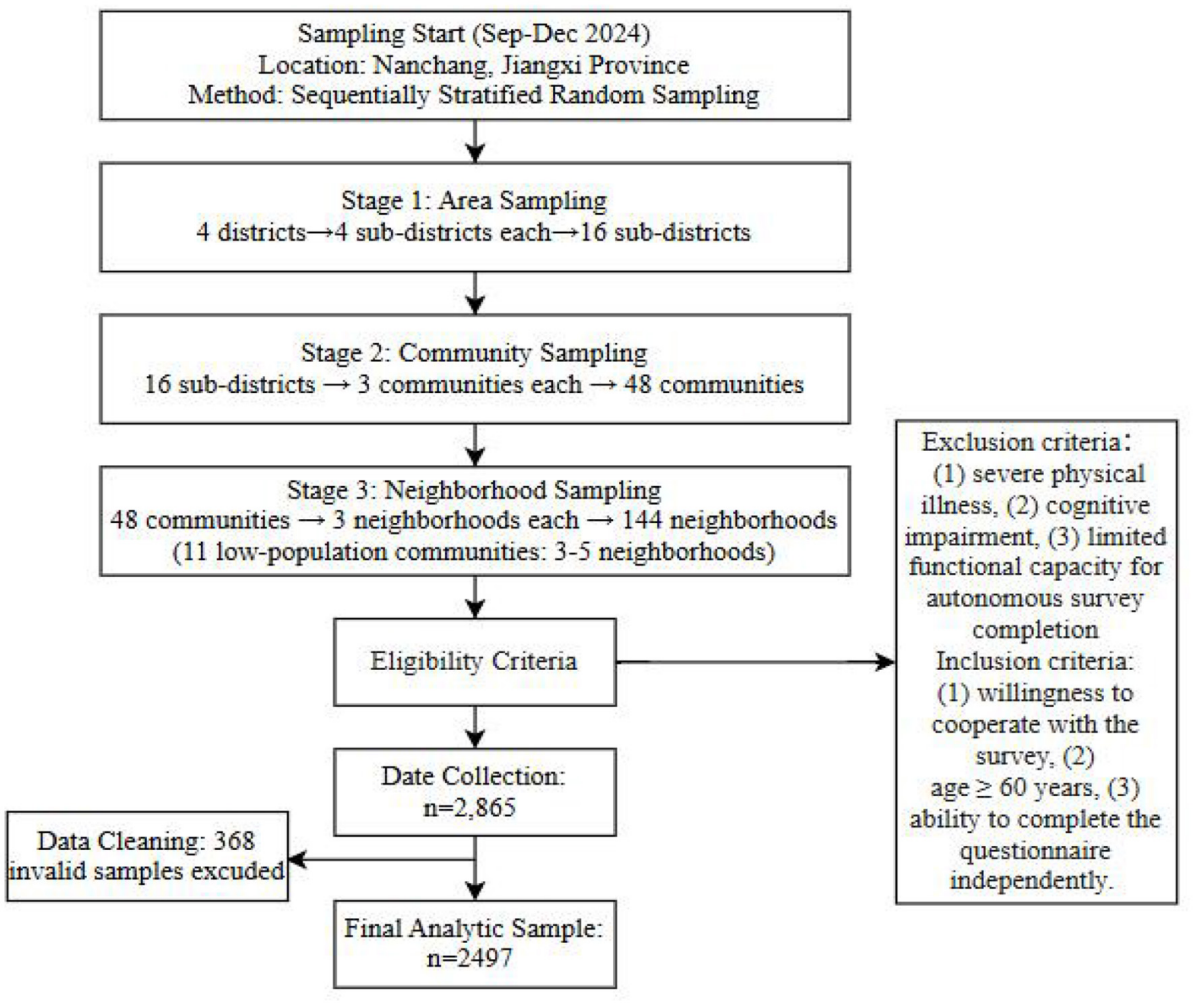
Flowchart of the sampling procedure.

All data are derived from offline surveys conducted in Nanchang City, Jiangxi Province, China, from September to December 2024. Methodologically, we implemented a sequentially stratified random sampling approach across multiple recruitment phases. Four districts were selected for the survey, with four streets sampled from each district, totaling 16 streets. Second, three communities were chosen from each of the 16 sub-districts, and three residential areas were selected from each of the 48 communities, totaling 144 residential areas. Due to the small population in some residential areas, 11 communities selected 3–5 residential areas for the survey. Inclusion criteria: (1) willingness to cooperate with the survey, (2) age ≥ 60 years, (3) ability to complete the questionnaire independently. Exclusion criteria: (1) severe physical illness, (2) cognitive impairment, (3) limited functional capacity for autonomous survey completion. Written informed consent was obtained from all enrolled participants prior to data collection. A total of 2,865 samples were collected, with 368 invalid samples excluded. The final analytic cohort comprised 2,497 eligible participants, representing an 87.43% valid response rate (Figure 1). There were 1,034 males (41.50%) and 1,463 females (58.50%); 1,906 were married (76.30%), 446 were widowed (18.70%), and 145 were divorced or other (5.00%); Educational stratification within the cohort revealed, elementary-level attainment or below predominated (*n* = 995; 40.50%), followed by junior secondary education (*n* = 1,125; 45.10%), with high school (*n* = 228; 9.10%) and tertiary qualifications (*n* = 129; 5.30%) comprising the remainder. This study was conducted with the approval of the Ethics Committee of Jiangxi Normal University (Approval No. IRB-JXNU-PEC-20240516) and in accordance with the principles of the Declaration of Helsinki.

### 2.2 Measures

#### 2.2.1 Physical activity rating scale (PARS-3)

This investigation employed Liang's revised Physical Activity Assessment Instrument (31) to quantify PA engagement among older adults. The tool evaluates three dimensions through Likert-type items (e.g., “Rate your typical exercise intensity”), utilizing a 5-point metric (1 = minimal, 5 = extensive). A composite score is derived from exercise intensity, duration, and frequency domains, with elevated scores indicating greater PA involvement. Using established stratification thresholds: scores <19 denote low PA, 20–42 moderate PA, and ≥43 high PA. The instrument demonstrated robust internal consistency (α=0.845).

#### 2.2.2 Pittsburgh sleep quality index (PSQI)

Sleep quality was evaluated using the Pittsburgh Sleep Quality Index (PSQI) (32), a validated instrument developed by Buysse (32). This 19-item tool assesses seven sleep domains through Likert-scaled responses (0–3), incorporating queries such as “Report your typical bedtime during the past month (24-h format)”. Elevated global scores denote superior sleep quality. Psychometric analysis confirmed robust internal consistency (α = 0.892).

#### 2.2.3 Patient health questionnaire-9 items (PHQ-9)

Depressive symptomatology was assessed using the 9-item Patient Health Questionnaire (PHQ-9) (33). This instrument evaluates symptom frequency across nine domains (e.g., “Experiencing self-critical thoughts or perceptions of personal/familial failure”) via a four-tiered frequency metric (0 = never; 3 = nearly daily). A threshold score of ≥5 indicates clinically significant depression, with escalating scores reflecting greater symptom severity. Psychometric validation demonstrated exceptional internal consistency (α= 0.941).

#### 2.2.4 Generalized anxiety disorder-7 (GAD-7)

Anxiety symptoms were evaluated using the Generalized Anxiety Disorder 7-item scale (GAD-7) (34). This instrument measures symptom frequency across seven domains (e.g., “Experiencing persistent nervous tension or irritability”) via a 4-point severity metric (0 = not at all; 3 = nearly every day). Clinical severity stratification delineates: 0–4 = minimal anxiety, 5–9 = mild, 10–13 = moderate, 14-18 = moderately severe, and 19–21 = severe anxiety. Elevated total scores correspond to greater symptom burden. Psychometric analysis confirmed robust internal consistency (α = 0.841).

### 2.3 Data analysis

Analyses were conducted using SPSS version 26.0 following a sequential analytical protocol. After performing descriptive characterization of demographic variables, sex differences in physical activity (PA), sleep quality, depression, and anxiety among older adults were assessed using chi-square tests. Subsequent analyses entailed adjustment for potential confounders, including gender, age, marital status, educational level, primary income source, number of chronic diseases, and self-rated health status. Separate logistic regression models were fitted to examine the independent associations of PA and sleep with depression and anxiety, respectively. Finally, multiplicative interaction terms between PA and sleep were introduced to evaluate their synergistic effects on depression and anxiety outcomes, with statistical significance defined as *P* < 0.05.

## 3 Results

### 3.1 Testing for common method bias

To address potential common method variance arising from the self-report nature of the measures, Harman's single-factor test was performed in accordance with established methodology (35). An exploratory factor analysis including all items related to physical activity, sleep quality, depression, and anxiety revealed four factors with eigenvalues above 1.0. The most substantial factor explained 23.391% of the total variance (<40%), suggesting that common method bias does not pose a significant threat to the interpretation of the results in this study. Suggesting minimal common method bias concern in this investigation.

### 3.2 Demographic information on older adults

The study cohort comprised 2,497 older adults (Table 1), with a gender distribution of 1,034 males (41.40%) and 1,463 females (58.60%). Physical activity stratification revealed low levels in 1,827 participants (73.20%), moderate in 422 (16.90%), and high in 248 (9.90%). Suboptimal sleep quality was observed in 1,011 individuals (40.50%), while 1,486 (59.50%) reported good sleep parameters. Clinically significant depressive symptomatology was present in 582 participants (23.30%), while anxiety manifestations affected 1,122 (44.90%) (Table 2). A significant gender disparity was observed in physical activity levels (χ^2^ = 22.724, *P* < 0.001). Females demonstrated lower participation in moderate-to-high intensity activities and were overrepresented in low-activity categories compared to males. Females exhibited substantially higher depression prevalence (390/1,463, 26.70% vs. 192/1,034, 18.6% in males; χ^2^ = 22.175, *P* < 0.001) and anxiety burden (731/1,463, 50.00% vs. 391/1,034, 37.80% in males; χ^2^ = 36.153, *P* < 0.01) (Table 3).

**Table 1:** Comparative analysis of physical activity and sleep quality relative to depression and anxiety across demographic subgroups in older adults (*N* = 2,497)

**Variable**	**Category**	***n* (%)**	**PA**	**PSQI**	**PHQ-9**	**GAD-7**
Age	60–69	1,218 (48.80%)	18.62 ± 28.40	4.67 ± 5.12	4.13 ± 5.27	4.62 ± 4.68
	70–79	914 (36.60%)	13.85 ± 23.90	7.01 ± 5.97	6.48 ± 7.27	6.71 ± 5.18
	>80	365 (14.60%)	11.30 ± 22.89	9.30 ± 5.434	6.30 ± 7.09	9.04 ± 4.38
	*F*		15.14^***^	116.623^***^	40.98^***^	132.35^***^
Marital status	Married	1,906 (76.30%)	15.69 ± 25.63	5.58 ± 5.64	5.28 ± 6.60	5.45 ± 5.04
	Bereavement	446 (17.90%)	16.52 ± 27.41	7.94 ± 5.70	5.57 ± 6.12	7.77 ± 4.71
	Divorce or other	145 (5.80%)	15.14 ± 29.81	8.90 ± 5.19	4.88 ± 5.25	8.25 ± 4.65
	*F*		0.23	49.31^***^	0.69	54.91^***^
Education	Primary school	955 (38.20%)	15.24 ± 24.81	6.03 ± 5.76	5.25 ± 6.35	5.87 ± 5.00
	Middle school	1,125 (45.10%)	17.20 ± 28.00	5.47 ± 5.62	5.00 ± 6.41	5.28 ± 5.00
	High school	288 (11.50%)	11.58 ± 20.96	9.19 ± 5.31	5.70 ± 6.38	8.94 ± 4.40
	College degree	129 (5.20%)	17.27 ± 29.71	9.07 ± 5.31	7.59 ± 7.12	7.31 ± 5.16
	*F*		3.85^**^	34.90^***^	6.74^***^	45.23^***^
Source of income	Oneself	1,627 (65.20%)	18.64 ± 28.09	4.97 ± 5.57	4.65 ± 6.03	4.59 ± 4.76
	Spouse	352 (14.10%)	11.51 ± 22.45	8.18 ± 5.59	6.51 ± 7.11	8.37 ± 4.63
	Children	393 (15.70%)	11.53 ± 22.54	8.07 ± 5.47	6.28 ± 7.02	8.36 ± 4.72
	Others	125 (5.00%)	4.39 ± 9.79	10.71 ± 3.17	7.53 ± 6.52	10.79 ± 2.60
	*F*		21.44^***^	86.83^***^	18.01^***^	158.08^***^
Number of chronic diseases	None	1,193 (47.80%)	18.80 ± 28.73	5.33 ± 5.61	4.88 ± 6.32	5.21 ± 5.01
	1 type	973 (39.00%)	15.29 ± 25.30	6.07 ± 5.71	5.49 ± 6.53	5.70 ± 5.01
	Two or more types	331 (13.20%)	6.52 ± 14.43	9.72 ± 4.88	6.32 ± 6.51	9.95 ± 3.43
	*F*		29.44^***^	81.35^***^	7.14^***^	128.63^***^
Self-assessment of physical condition	Better	758 (30.40%)	13.96 ± 23.88	6.66 ± 5.83	5.34 ± 6.61	6.61 ± 5.09
	Generally	1,309 (52.40%)	20.33 ± 29.59	5.05 ± 5.57	4.95 ± 6.24	4.73 ± 4.87
	Poor	430 (17.20%)	5.30 ± 11.87	8.85 ± 5.03	6.36 ± 6.67	8.95 ± 4.16
	*F*		58.51^***^	79.38^***^	7.82^***^	131.12^***^

PSQI, Pittsburgh Sleep Quality Index; GAD-7, Generalized Anxiety Disorder-7; PA, Physical Activity Rating Scale; PHQ-9, Patient Health Questionnaire-9 items. ^*^P < 0.05, ^**^P < 0.01, and ^***^P < 0.001. Same below.

**Table 2:** Factor-specific characterization of physical activity, sleep quality, depression, and anxiety in sex-stratified geriatric cohorts.

**Gender**	**Category**	**Man**	**Woman**	**Total**	**χ^2^**	***P***
*N*		1,034 (41.40%)	1,463 (58.60%)	2,497		
PA	Low	705 (68.20%)	1,122 (76.70%)	1,827 (73.20%)	22.724	< 0.001
	Medium	211 (20.40%)	211 (14.40%)	422 (16.90%)		
	High	118 (11.40%)	130 (8.90%)	248 (9.90%)		
PSQI	Poor	400 (38.70%)	611 (41.80%)	1,011 (40.50%)	2.383	>0.05
	Good	634 (61.30%)	852 (58.20%)	1,486 (59.50%)		
PHQ-9	No	842 (81.40%)	1,073 (73.30%)	1,915 (76.70%)	22.175	< 0.001
	Yes	192 (18.60%)	390 (26.70%)	582 (23.30%)		
GAD-7	No	643 (62.20%)	732 (50.00%)	1,375 (55.10%)	36.153	< 0.001
	Yes	391 (37.80%)	731 (50.00%)	1,122 (44.90%)		

**Table 3:** Binary logistic regression analysis of the interaction between physical activity, sleep quality, depression, and anxiety.

**Independent variable**	**PHQ-9**				**GAD-7**			
	***P***	**Exp (B)**	**95% CI**		***P***	**Exp (B)**	**95% CI**	
			**LLCI**	**ULCI**			**LLCI**	**ULCI**
Low physical activity × Poor sleep quality		1.00				1.00		
Low physical activity × Good sleep quality	0.001	0.33	0.26	0.42	0.001	0.11	0.09	0.15
Moderate physical activity × poor sleep quality	0.010	0.49	0.29	0.81	0.850	1.07	0.51	2.27
Moderate physical activity × Good sleep quality	0.001	0.03	0.01	0.10	0.001	0.05	0.03	0.09
High physical activity × poor sleep quality	0.001	0.29	0.12	0.67	0.020	0.37	0.16	0.84
High physical activity × Good sleep quality	0.001	0.01	0.01	0.04	0.001	0.02	0.01	0.03

### 3.3 The relationship between physical activity, sleep quality, depression, and anxiety in older adults

Following adjustment for core demographic covariates in the older adult population, multivariable logistic regression models were specified with depression/anxiety dichotomization (reference: absence) as outcomes. Primary exposures included physical activity (reference: high PA) and sleep quality (reference: good sleep). The analysis revealed significantly elevated depression risk with low PA (OR = 9.46, 95%CI 2.89–30.97) and anxiety vulnerability (OR = 3.66, 95%CI 2.21–6.06). Moderate PA independently predicted anxiety (OR = 2.85, 95%CI 1.49–5.46). Suboptimal sleep quality demonstrated strong associations with both depression (OR = 2.40, 95%CI 1.40–4.12) and anxiety (OR = 9.96, 95%CI 7.34–13.52), with all associations statistically significant (*P* < 0.05).

### 3.4 The interaction between physical activity and sleep quality and its relationship with depression and anxiety

Before examining the interaction effects, we assessed collinearity among the measures of physical activity, sleep quality, depression, and anxiety. Variance inflation factors ranged from 1.128 to 1.614, well below the conservative threshold of 3.0, indicating no multicollinearity and meeting the assumptions for subsequent modeling. Subsequent model fit assessment yielded excellent indices: normed χ^2^*/df* = 2.189, RMSEA = 0.022, CFI = 0.990, NFI = 0.982, RFI = 0.980, TLI = 0.989, and GFI = 0.990. All metrics exceeded recognized thresholds for model fit, demonstrating that the model is well specified and has sufficient statistical power to test interaction effects.

**Figure 2:**
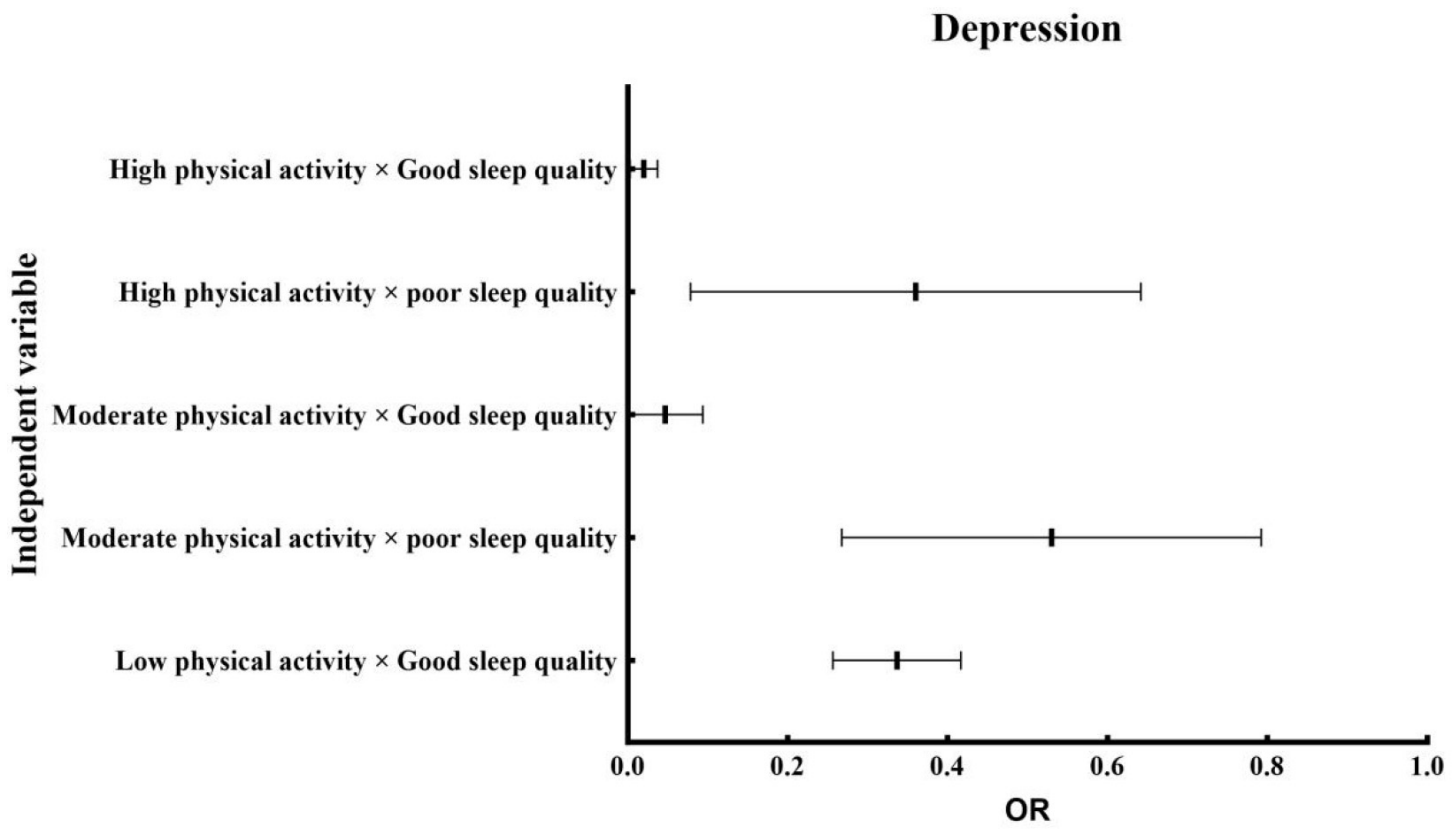
Depression forest plot.

**Figure 3:**
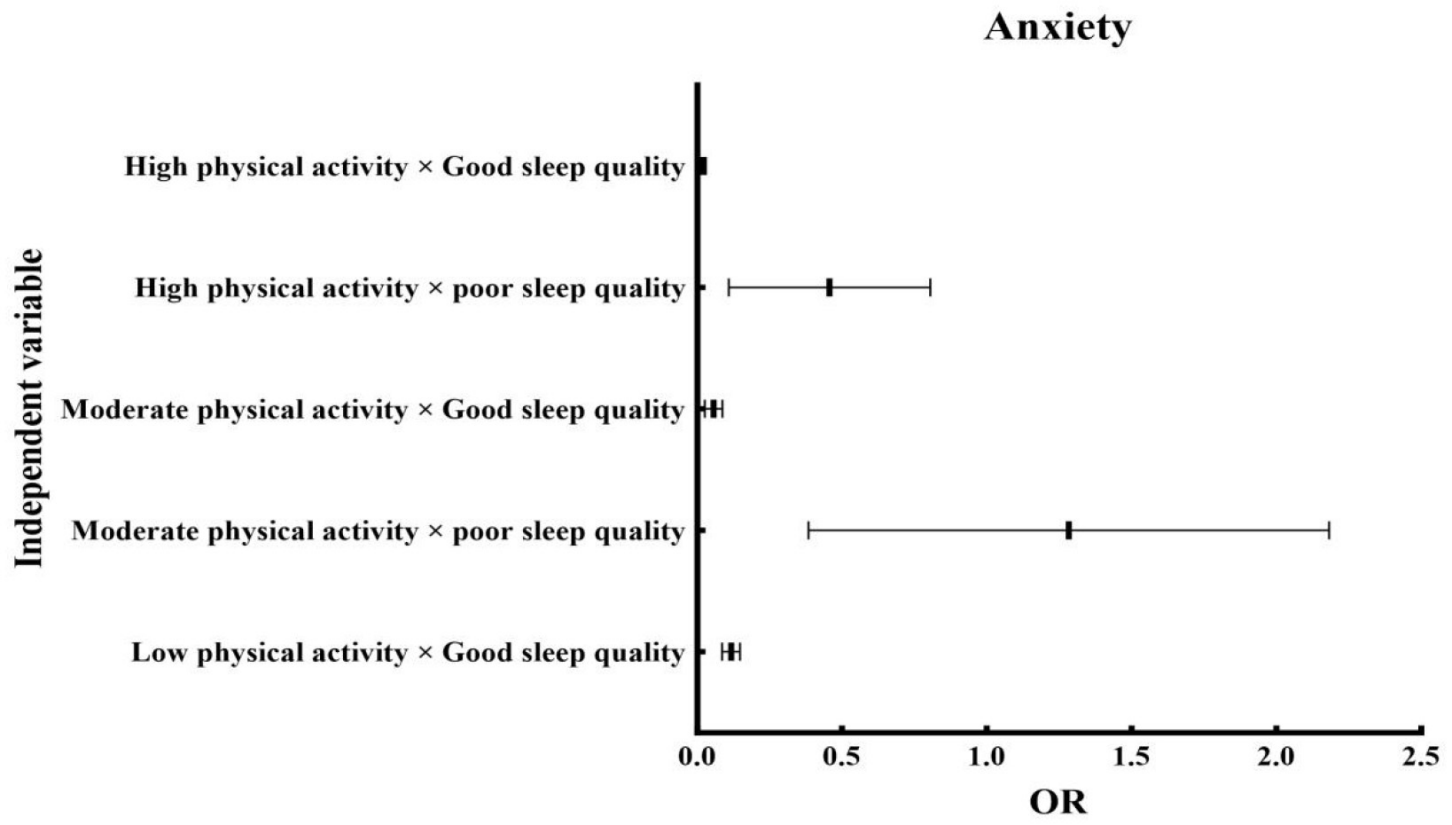
Anxiety forest plot.

Following adjustment for core demographic covariates (Table 4), binary logistic regression models examined PA-sleep interaction effects (reference: low PA × poor sleep) on depression and anxiety outcomes (Figures 2, 3). Analyses revealed significant effects between physical activity and sleep quality on modulating affective symptomatology (depression, anxiety)in older adults. Relative to the reference group (low PA/poor sleep), Individuals with good sleep quality demonstrated significantly reduced depression/anxiety across all PA levels. Those with poor sleep showed depression reduction regardless of PA status. High PA with poor sleep conferred specific anxiety reduction (*P* < 0.05). Notably, no significant anxiety and PA interaction was found in the moderate PA and poor sleep subgroup (*P* > 0.05).

**Table 4:** Binary logistic regression analysis of covariates on depression and anxiety.

**Control variable**	**PHQ-9**				**GAD-7**			
	**P**	**Exp (B)**	**95% CI**		**P**	**Exp (B)**	**95% CI**	
			**LLCI**	**ULCI**			**LLCI**	**ULCI**
Gender	0.001	1.56	1.27	1.93	0.001	2.53	1.96	3.26
Age	0.001	1.27	1.11	1.47	0.001	2.95	2.45	3.55
Marital status	0.116	0.87	0.72	1.04	0.001	1.79	1.40	2.30
Education	0.001	1.25	1.10	1.40	0.001	1.29	1.11	1.51
Source of income	0.001	1.22	1.90	1.36	0.001	2.01	1.73	2.34
Number of chronic diseases	0.851	1.01	0.88	1.17	0.001	1.79	1.50	2.15
Self-assessment of physical condition	0.670	1.00	0.90	1.19	0.056	1.18	1.00	1.41

## 4 Discussion

In this survey of older adults over the age of 60 living in the community, 73.20% (*n* = 827) exhibited low PA. This rate is slightly lower than the 78.7% reported by Wang et al. (36) but remains markedly higher than the 60.9% documented among older adults in the United States (37). Moreover, Males demonstrated significantly higher PA metrics than females (38–40). Previous research reflects higher activity frequency among older women but greater intensity among men (41). Meta-analytic evidence indicates male preference for vigorous pursuits (golf, bowling, resistance training, team sports) vs. female proclivity for lower-intensity activities (walking, domestic tasks, retail activities, community dance) (42). Positive self-rated health correlated strongly with elevated PA engagement (43, 44). Substantiating the perception-activity feedback loop. Concurrently, 40.50% of participants exhibited clinically significant sleep disturbances, a proportion higher than the 35.9% pooled estimate reported by Lu et al. (45). aligning with multinational prevalence studies (46–48). Older adult females demonstrated 2.3-fold higher sleep disorder prevalence than males, potentially attributable to shortened endogenous circadian periodicity that heightens vulnerability to chronodisruption. In addition, the detection rate of depression and anxiety is higher in older women than in older men. Previous studies have also confirmed this (49–51). Affective disorder prevalence was markedly elevated in females (depression: 26.70% vs. 18.60%; anxiety: 50.00% vs. 37.80%). This disparity may stem from sexually dimorphic neurochemical susceptibility (52–54). Age-related decline in monoaminergic neurotransmission, particularly within serotonergic and dopaminergic systems, disproportionately disrupts affective regulation in females. This effect is mediated through several neurobiological mechanisms, including an accelerated rate of neurotransmitter catabolism, estrogen-mediated alterations in receptor sensitivity, and diminished neurotrophic support within limbic structures (55, 56).

Multivariable regression analyses demonstrated that physical activity (PA) and sleep quality function as protective factors, mitigating depression and anxiety incidence risk among older adults after demographic adjustment. The biopsychosocial framework (57) underscores biological, psychological, and social determinants in ameliorating geriatric affective disturbances. Physiologically, exercise potentiates monoamine neurotransmission (58) and chronically attenuates hypothalamic-pituitary-adrenal (HPA) axis hyperactivity (59, 60). thereby reducing allostatic load and improving affective symptomatology. Psychologically, moderate PA facilitates Stress dysregulation mitigation (61), improves psychological resilience (62, 63), and Self-efficacy fortification (64, 65). it can promote interpersonal communication and increase social interaction (66, 67) and collectively foster affective disorder resilience (68). Comparatively, suboptimal sleep elevates depression risk 2.4-fold and anxiety vulnerability 9.96-fold vs. optimal sleepers, indicating that superior sleep parameters substantially reduce Depression and anxiety risk (69). Empirical observations confirm elevated depression prevalence in sleep-disordered populations vs. general cohorts (27, 70). Establishing sleep quality as a cardinal modulator of affective disorder susceptibility. Crucially, comparative effect magnitude analysis reveals sleep quality demonstrates superior protective effect sizes against depression/anxiety relative to PA, constituting the principal etiological precursor of geriatric affective pathology.

Binary logistic regression revealed significant PA-sleep interaction effects on depression and anxiety in older adults. Relative to the reference group (low PA/poor sleep), Individuals with good sleep quality demonstrated reduced depression/anxiety across all PA levels. Those maintaining PA engagement despite poor sleep showed depression risk mitigation. High PA coupled with poor sleep conferred specific anxiety reduction. These patterns indicate that optimizing either PA or sleep quality provides effective risk mitigation when both factors are suboptimal. Empirical evidence supports the existence of bidirectional neuroregulatory pathways linking physical activity and sleep (71–73), which collectively modulate emotional states in older adults. Mechanistically, PA stimulates monoamine neurotransmission essential for mood regulation (74). Quality sleep maintains neurochemical homeostasis, enhancing daytime neuromodulatory efficiency. Furthermore, regular PA potentiates sleep architecture through shortened sleep latency and extended sleep duration (75, 76). Conversely, restorative sleep (77, 78) enhances exercise capacity via Improved physiological recovery, Increased exercise endurance, and Optimal energy mobilization. In turn, provides better recovery and preparation for physical activity, giving older adults more energy and endurance during exercise. This mutually beneficial relationship helps older adults develop healthy lifestyles, effectively diminishing population-attributable risk for mood and anxiety disorders (79, 80).

### 4.1 Limitations

This investigation acknowledges several limitations regarding generalizability. First, the geographically circumscribed sampling frame, which was exclusively recruited from an urban cohort in Nanchang, China, may constrain extrapolation to broader geriatric populations. Region-specific socio-ecological covariates such as healthcare accessibility and cultural practices potentially modulate physical activity (PA), sleep architecture, and affective outcomes. Subsequent research should therefore implement stratified random sampling across heterogeneous settings, such as rural, suburban, and multi-center locations, while incorporating socio-ecological covariates like ethnic composition within covariate-adjusted models to enhance ecological validity.

Second, the self-report methodology precludes definitive causal attribution regarding physical activity and sleep quality on affective disorders. Crucially, the temporal precedence remains indeterminate; it is unclear whether PA enhancement drives sleep improvement or, conversely, superior sleep facilitates PA engagement. To address this, subsequent research will implement prospective sequential designs, including randomized controlled trials (RCTs) with protocolized PA and sleep interventions along with multi-wave assessments. These will integrate time-lagged analytics and cross-lagged structural equation modeling to elucidate directional pathways among PA parameters, sleep architecture, and mental health trajectories across temporal domains.

Third, a major limitation concerns the pervasive use of self-reported measures across all constructs, including physical activity, sleep quality, depression, and anxiety. Such exclusive reliance on subjective reports introduces substantial risks of recall bias, social desirability bias, and cognitive interpretation errors. These biases may be especially pronounced among older adults, who may exhibit varying levels of neurocognitive integrity. The absence of objective measures, such as accelerometry-based physical activity monitoring, polysomnographic sleep assessment, or clinician-administered diagnostic interviews, considerably limits the accuracy and robustness of the data. Future studies should incorporate multimodal assessment strategies that combine self-report with objective metrics to strengthen validity and reduce measurement bias.

## 5 Conclusions

A substantial proportion of older adults exhibited insufficient physical activity (PA) levels. Notably, older women demonstrated poorer sleep quality and more severe depressive and anxiety symptoms compared to their male counterparts. Critically, the interaction between PA and sleep quality exerted significant effects on geriatric depression and anxiety. These findings confirm that both PA engagement and sleep optimization constitute effective non-pharmacological interventions for mitigating affective disorders in aging populations. First, Governments and community centers should implement age-appropriate exercise initiatives (e.g., group walking programs, Tai Chi classes) specifically designed for older adults. Multimodal interventions combining sleep hygiene education (e.g., circadian rhythm management, sleep environment optimization) with mental health workshops should be delivered through community health campaigns. However, it should be acknowledged that the generalizability of these findings may be constrained to urban contexts within China.

## Data Availability

The original contributions presented in the study are included in the article/supplementary material, further inquiries can be directed to the corresponding authors.
